# The Absence of CCR7 Results in Dysregulated Monocyte Migration and Immunosuppression Facilitating Chronic Cutaneous Leishmaniasis

**DOI:** 10.1371/journal.pone.0079098

**Published:** 2013-10-30

**Authors:** Jessica C. Kling, Matthias Mack, Heinrich Körner

**Affiliations:** 1 Menzies Research Institute, Hobart, Tasmania, Australia; 2 Innere Medizin II, Nephrologie/Forschung, Universitätsklinikum Regensburg, Regensburg, Germany; Federal Institute for Vaccines and Biomedicines, Germany

## Abstract

The protozoan parasite *Leishmania major* causes cutaneous lesions to develop at the site of infection, which are resolved with a strong Th1 immune response in resistant hosts, such as C57BL/6 mice. In contrast, the lesions ulcerate in susceptible hosts which display a Th2 response, such as BALB/c mice. The migration of cells in the immune response to *L. major* is regulated by chemokines and their receptors. The chemokine receptor CCR7 is expressed on activated DCs and naïve T cells, allowing them to migrate to the correct micro-anatomical positions within secondary lymphoid organs. While there have been many studies on the function of CCR7 during homeostasis or using model antigens, there are very few studies on the role of CCR7 during infection. In this study, we show that B6.CCR7^-/-^ mice were unable to resolve the lesion and developed a chronic disease. The composition of the local infiltrate at the lesion was significantly skewed toward neutrophils while the proportion of CCR2^+^ monocytes was reduced. Furthermore, a greater percentage of CCR2^+^ monocytes expressed CCR7 in the footpad than in the lymph node or spleen of B6.WT mice. We also found an increased percentage of regulatory T cells in the draining lymph node of B6.CCR7^-/-^ mice throughout infection. Additionally, the cytokine milieu of the lymph node showed a Th2 bias, rather than the resistant Th1 phenotype. This data shows that CCR7 is required for a protective immune response to intracellular *L. major* infection.

## Introduction

The efficient movement of cells during ontogenesis, homeostatic recirculation and inflammatory recruitment is governed by a family of G protein-coupled seven-transmembrane spanning receptors that specifically bind to small chemotactic cytokines termed chemokines [1,2]. In the mammalian immune system, the navigation of cell subsets is facilitated by distinct expression patterns of chemokine receptors on the cell surface and the presentation of their ligands in the form of fixed gradients in peripheral and lymphoid tissues [3]. Ultimately, this targeted movement is central for the induction of tolerance and the generation of a protective immune response [4]. 

One of the most important events in the generation of a cellular immune response is the encounter of antigen-specific naïve T cells with antigen presenting cells. Antigen presentation occurs in the T cell zone of secondary lymphoid organs, such as lymph nodes and spleen, under the guidance of an array of chemokine receptors and their ligands [5] and results in the differentiation of T cells into a range of effector phenotypes [6]. However, one receptor, CC-chemokine receptor 7 (CCR7) which is expressed on naïve and memory T cells, and in addition, on monocytes and antigen-presenting cells, has been described to play a dominant role in this process [7]. Despite the seemingly central function of this chemokine receptor, protective immune responses have been reported in a variety of CCR7-deficient models of viral [8,9] and bacterial infection [10], and it has been discussed that the magnitude of the role of CCR7 is inversely proportional to the amount of antigen that initiates the response [10].

While cutaneous leishmaniasis has been studied in a model with transgenically decreased CCR7 expression, toxoplasmosis is the only parasitic model investigated to date in the CCR7^-/-^ mouse model, and this model shows a high degree of susceptibility [11,12]. Therefore, we decided to test the role of CCR7 in a gene-deficient model during experimental cutaneous leishmaniasis. Under normal circumstances, the infection is confined to the skin and local draining lymph node, and is resolved quickly in immunocompetent hosts such as C57BL/6 mice, but can spread to the visceral organs and progress to a fatal outcome in immunocompromised hosts [13]. The analysis of *L. major* infected B6.CCR7^-/-^ mice showed an influence of this receptor on the recruitment of monocytes, the regulation of the adaptive immune response and the local cytokine milieu in the draining lymph node. Taken together, these alterations contribute to a failure to resolve the infection completely and consequently the mice develop a chronic disease.

## Materials and Methods

### Mice

Genetically targeted C57BL/6 mice deficient for CCR7 (B6.CCR7^-/-^, B6.129P2(C)-*Ccr7^tm1Rfor^*/J, [14]) and control C57BL/6J (B6.WT) mice were obtained from Jackson Laboratory (Bar Harbor, USA). The mice were screened according to the previously published protocol [14]. All animals were kept under specific pathogen free conditions at the animal research facilities of the Comparative Genomics Centre, James Cook University, Townsville, Australia and the Menzies Research Institute Tasmania, Hobart, Australia. Mice of both sexes were infected at 8-12 weeks of age.

### Ethics Statement

The experiments followed protocols approved by the animal ethics committees of James Cook University, Townsville under ethics numbers A1170 and A1492, and the University of Tasmania, Hobart under ethics number A11656.

### Leishmania

The pathogenic parasite substrains *L. major* BNI (MHOM/IL/81/FE/BNI) and *L. major* Friedlin expressing eGFP (MHOM/IL/80/Friedlin-eGFP) were maintained and expanded in supplemented Schneider’s media (Life Technologies, Mulgrave, Australia) essentially as published [15]. Immediately prior to infection, parasites were passaged onto rabbit blood agar plates in supplemented RPMI media for 6-8 days as described [15]. To maintain infectivity, the parasites were passaged through BALB/c mice. Mice were infected subcutaneously with 3x10^6^ *L. major* promastigotes in the hind footpad. Footpad lesions were measured using a metric caliper to determine disease progression as described [16].

### Flow cytometry

Tissues were harvested from infected footpads, draining popliteal lymph nodes and spleens. Leukocytes were isolated by mechanical disruption of the tissue and were restimulated with phorbol 12-myristate 13-acetate (PMA; 20 ng/mL), ionomycin (1 μM) and protein transport inhibitor (monensin; BD GolgiStop) for four to six hours. Multi-colour flow cytometry was performed following a three-step protocol. First, cells were blocked for unspecific Fc binding with rat anti-mouse CD16/32 (clone 93; eBioscience, San Diego, USA). Secondly, cells were stained for surface markers: rat anti-mouse Ly6C (FITC; clone AL-21; BD Pharmingen), rat anti-mouse Ly6G (PE; clone 1A8; BD Pharmingen), rat anti-mouse CD11b (PerCP-Cy5.5; clone M1/70; BD Pharmingen), Armenian hamster anti-mouse CD11c (APC; clone HL3; BD Pharmingen), rat anti-mouse CD4 (PerCP-Cy5.5; clone RM4-5; BD Pharmingen), rat anti-mouse CD8a (Pacific Blue; clone 53-6.7; BD Pharmingen) and rat anti-mouse CD25 (APC-Cy7 or PE; clone PC61; BD Pharmingen). The rat anti-mouse CCR2 antibody (unlabelled, clone MC-21) was provided by M. Mack, Regensburg, Germany [17]. The cells were then fixed according to the manufacturer’s instructions with FoxP3 Fix/Perm buffer (BioLegend, San Diego, USA) and permeabilized with either Perm buffer (BioLegend) or saponin (Sigma-Aldrich, Sydney, Australia) in PBS. In the third step, the cells were stained intracellularly with rat anti-mouse IL-17 (PE; clone TC11-18H10; BD Pharmingen), rat anti-mouse/rat FoxP3 (APC; clone FJK-16s; eBioscience), rat anti-mouse IL-10 (PE; clone JES5-16E3; BD Pharmingen), rat anti-mouse IFN-γ (Alexa Fluor 488; clone XMG1.2; BD Pharmingen), rat anti-mouse IL-12 (p40/p70) (APC; clone C15.6; BD Pharmingen) and rat anti-mouse IL-4 (PE; clone 11B11; BD Pharmingen). A polyclonal rabbit serum was used to detect mouse iNOS (pAb, BD Biosciences). Donkey anti-rat IgG (Dylight 649; Jackson Immunoresearch, West Grove, USA) and goat anti-rabbit IgG (Pacific Blue; Molecular Probes, Mulgrave, Australia) were used as secondary antibodies. Cells were acquired on a Beckman Coulter CyAn ADP flow cytometer using Summit version 4.3 and samples were analysed using FlowJo software (Tree Star, Inc.). Non-leukocytic populations in the footpad were excluded by forward and side scatter analysis.

### Cytometric bead array

Cytometric bead arrays (CBA) were performed on serum collected from the peripheral blood of infected mice. CBA flex sets for IFN-γ, IL-12p70, IL-1β, TNF, IL-4 and IL-10 (BD Biosciences) were used to determine cytokine levels and the manufacturer’s instructions were essentially followed. Samples were acquired on a BD FACSCanto II using FACSDiva 6.1 software and analysed with FCAP Array version 1.0 software.

### Limiting dilutions

Limiting dilution experiments were performed to determine the parasite burden in infected footpads and draining popliteal lymph nodes. Single cell suspensions were prepared in supplemented Schneider’s media and serial dilutions (5-fold) were pipetted across a 96-well plate with eight replicates for each organ in an end-point titration. The plates were incubated for 10-14 days at 27°C before the number of *Leishmania*-positive wells was determined under a light microscope. To calculate the parasite burden a generalised Pearson chi-square test was performed (L-Calc version 1.1). Alternatively, extreme limiting dilution analysis (ELDA) was used [18]. 

### Confocal Microscopy

Spleens and draining lymph nodes were isolated from B6.WT and B6.CCR7^-/-^ mice, rapidly frozen in Tissue Tek optimal cutting temperature (OCT) media (ProSciTech, Townsville, Australia) and stored at -80°C. Sections were cut using a cryotome (Leica, North Ryde, Australia), air-dried and fixed in acetone at -20°C. Prior to staining, sections were re-hydrated in PBS+1% BSA for 15 minutes and then permeabilised in TBS+0.1% Tween-20 for 15 minutes if intracellular markers were examined. 

Sections were stained using rat anti-mouse B220 (clone RA3-6B2; BD Pharmingen), rat anti-mouse CD11b (biotin; clone M1/70; BD Pharmingen), rat anti-mouse Ly6C (FITC; clone AL-21; BD Pharmingen), rat anti-mouse Ly6G (PE; clone 1A8; BD Pharmingen), mouse anti-mouse iNOS (FITC; clone 6/iNOS/NOS type II; BD Transduction Laboratories) and rabbit anti-*L. major* serum (Cy5; [19]). Secondary staining was performed using streptavidin conjugated to Alexa Fluor 546 or FITC (Life Technologies) and goat anti-rat IgG (Alexa Fluor 633; Life Technologies) before the sections were mounted with polyvinyl alcohol mounting media with DABCO (Sigma-Aldrich) to prevent fading and allowed to dry overnight. Images were acquired using a Zeiss LSM 510 confocal microscope.

### Blocking of IL-10R and IL-4 in vivo

Mice were injected intraperitoneally every second day, starting the day prior to *L. major* infection, with PBS, anti-IL-4 (clone 11B11, obtained from hybridoma supernatant) or anti-IL-10R (clone 1B1.3a, obtained from hybridoma supernatant). The draining popliteal lymph node was analysed at day 14 post infection by flow cytometry.

### Statistical analysis

A two-tailed Mann-Whitney test or an unpaired t test was performed using GraphPad Prism 5 for Mac OS X, version 5.0a (GraphPad Software, San Diego, USA) to determine p values. A p value below 0.05 was considered statistically significant.

## Results

### Cutaneous leishmaniasis in B6.CCR7^-/-^ mice

**Figure 1:**
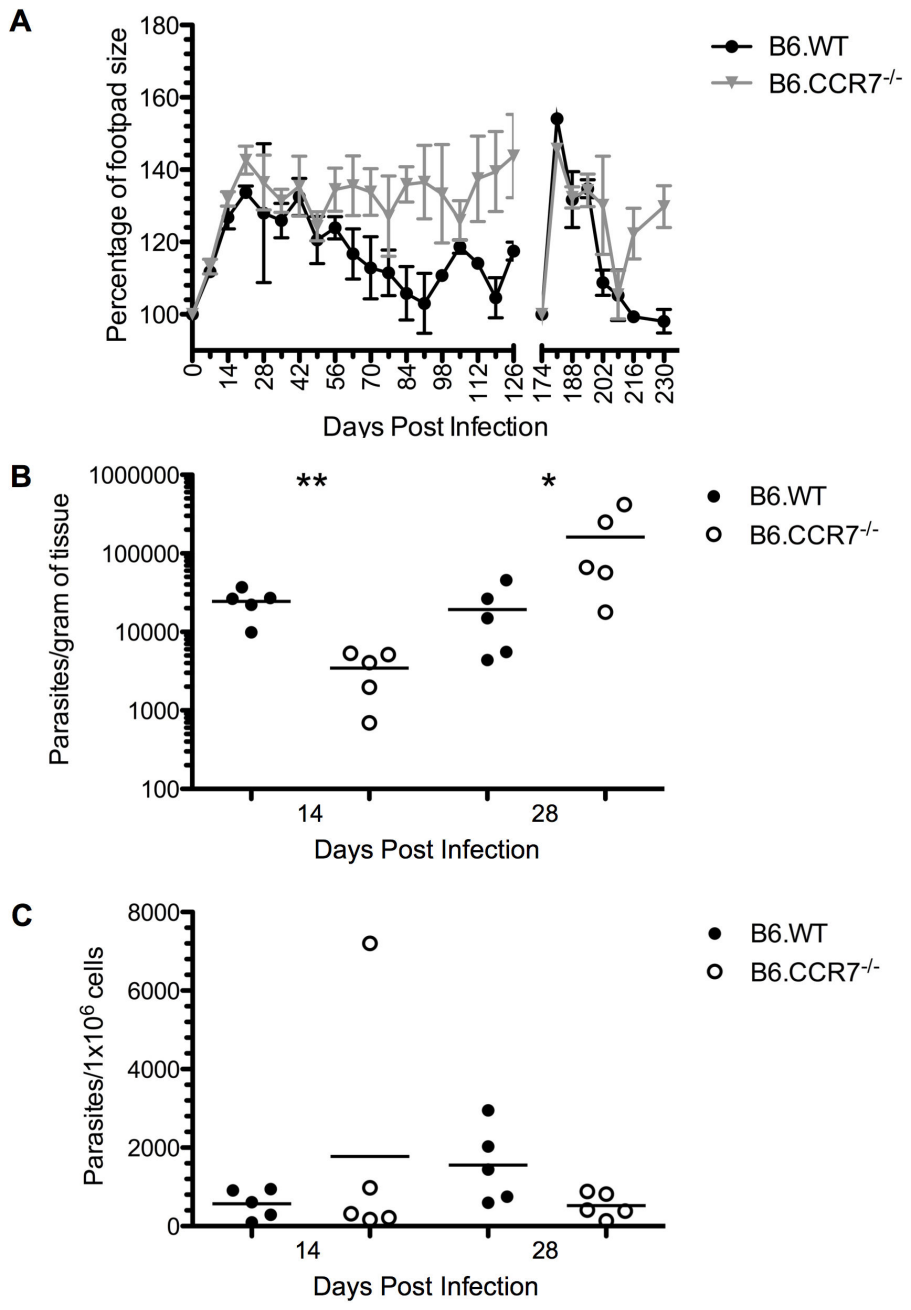
Disease progression in B6.CCR7^-/-^ mice after infection with *L. major*. (A) Mice were infected subcutaneously with *L. major* and the footpad lesion was measured weekly with a metric caliper. The lesion size was compared to the mean of the uninfected footpads from each experimental group (100%). Mice were infected subcutaneously in the contralateral footpad at day 174 post infection to measure the memory response. In accordance with animal ethics guidelines to minimise the usage of mice, this experiment was conducted at the same time as previously published data, and as a result, the lesion size of B6.WT mice has been published [61]. The parasite burdens in the footpad (B) and draining lymph node (C) were determined by limiting dilutions at days 14 and 28 post infection for B6.WT (closed circles) and B6.CCR7^-/-^ (open circles) mice. One representative analysis of two experiments; Experimental group size: n=5 mice/genotype; *p<0.05; **p<0.01.

Subcutaneous infection of the footpad with *L. major* metacyclic promastigotes causes a lesion that is typically resolved with little scarring within 8-10 weeks in B6.WT mice [16]. In the infection of B6.WT and B6.CCR7^-/-^ mice presented, we saw a two-phasic footpad swelling (peaks at day 21 and day 105) in the clinically healthy control group (Figure 1A). In contrast, B6.CCR7^-/-^ mice were unable to heal the lesion and developed a chronic infection resulting in a permanent swelling of the footpad (Figure 1A). Unlike *L. major* infection of susceptible BALB/c mice, the lesions of B6.CCR7^-/-^ mice did not ulcerate.

In the human immune system, CCR7 has been shown to be a marker for central memory cells [20], which generally home to secondary lymphoid organs. Therefore, we explored the possibility that this chemokine receptor facilitates an adaptive memory response to *L. major*. When B6.CCR7^-/-^ mice were reinfected in the contralateral footpad after 174 days, they first appeared to clear the infection similar to B6.WT mice, according to lesion size, indicating no major impact of CCR7 deficiency on memory formation (Figure 1A). Six weeks after reinfection, the response of the control B6.WT mice subsided and the lesion resolved, whereas the lesion size in B6.CCR7^-/-^ mice increased again and remained enlarged for an extended period of time. 

The parasite burden in the infected footpad and draining lymph node was quantified during the first four weeks of infection by performing limiting dilutions. At day 14, before the peak of infection, B6.CCR7^-/-^ mice initially controlled parasite replication in the footpad (Figure 1B), but by day 28 after infection, B6.CCR7^-/-^ mice had an increase in the number of parasites in the footpad as compared to B6.WT mice (161820 ± 75472 [mean ± SEM] and 19346 ± 7621, respectively, p=0.03). In contrast, we could not detect a significant difference in parasite numbers in the draining lymph nodes of the two genotypes (Figure 1C). We concluded that while B6.CCR7^-/-^ mice may be able to control parasite dissemination at an early stage of infection, there is a breakdown of the control, which allows parasites to replicate and cause a chronic disease in these mice.

In the mice reinfected for the analysis of the memory response (Figure 1A), the amount of parasites in the contralateral footpad was quantified. Although there were no detectable parasites in B6.WT mice, a substantial number could be found in the footpad tissue in B6.CCR7^-/-^ mice (149.1 ± 38.6 parasites/gram of tissue, n=3 mice), further demonstrating a role for CCR7 in control of the replication of parasites within the footpad tissue.

### Increased neutrophilic infiltrate at the expense of CCR2^+^ monocytes

**Figure 2:**
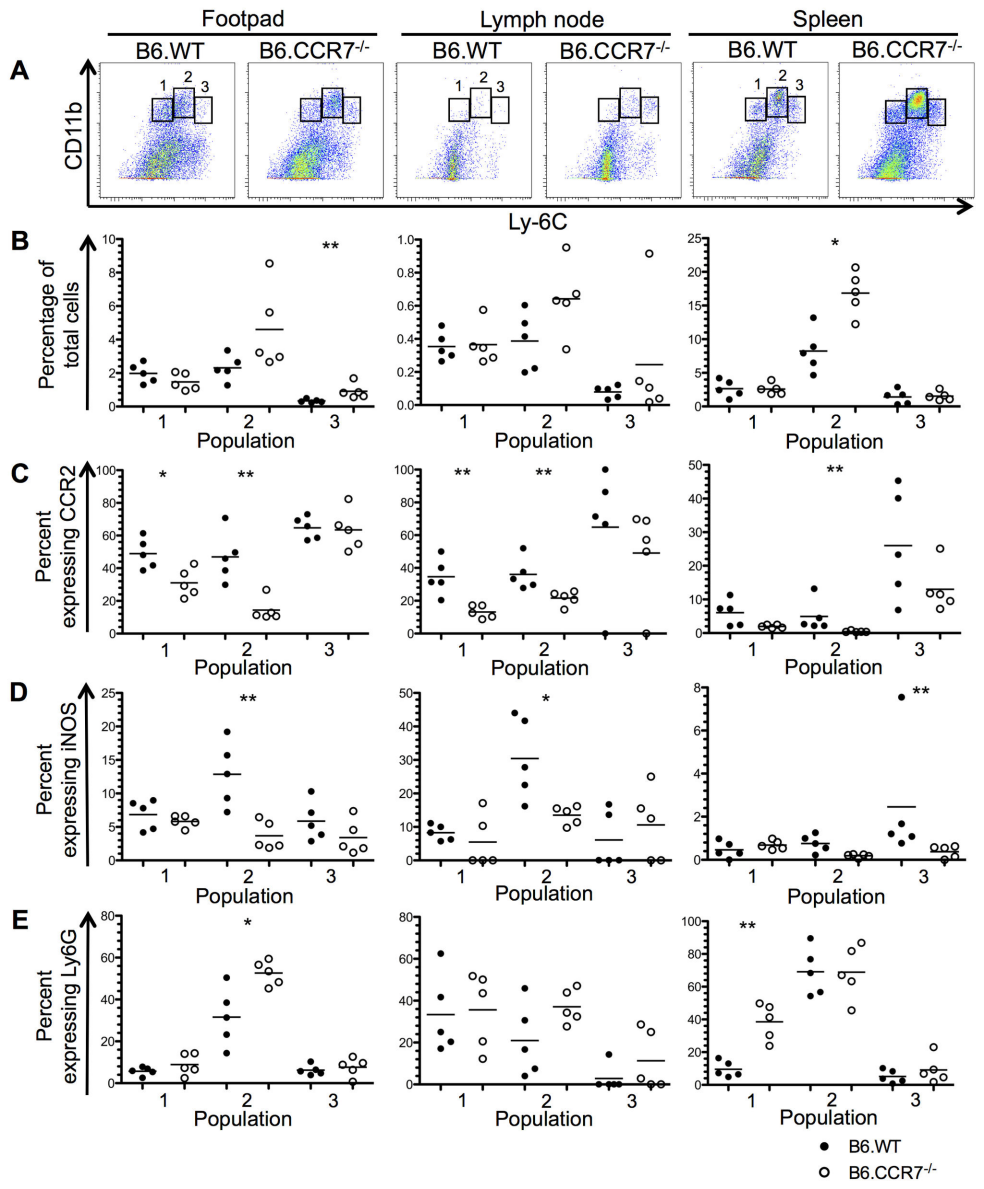
Monocytic populations in the footpad, lymph node and spleen at day 14 post infection. (A) CD11b^+^ monocytes from the footpad, lymph node and spleen were classified into different populations according to their Ly6C expression using flow cytometry. (B) The percentage of total cells recovered from each tissue in B6.WT (closed circles) and B6.CCR7^-/-^ (open circles) mice was determined for each population. The percentage of cells from each population expressing CCR2 (C), iNOS (D) and Ly6G (E) was determined by flow cytometry. One representative analysis of three experiments; Experimental group size: n=5 mice/genotype; *p<0.05; **p<0.01.

The monocytic and neutrophilic populations in the infected lesion, draining lymph node and spleen were analysed by flow cytometry at days 14, 28 and 42 after infection. These timepoints were chosen to analyse any early changes to the immune response (day 14) and any differences at the peak of infection (day 28). The disease progression between the two genotypes differentiates at day 42 after infection, so this timepoint was used to determine any factors that contribute to the chronic infection. Monocytes were identified using CD11b and further classified into various populations according to their Ly6C expression. These subpopulations were additionally analysed for their Ly6G, CCR2 and iNOS expression, and representative plots are shown in Figure S1. At day 14 after infection, three monocytic populations were defined in the lesion, draining lymph node and spleen (Figure 2A). The Ly6C^lo^ population (population 1) represented tissue resident monocytes as suggested by their low CCR2 and iNOS expression in the footpad and lymph node (Figure 2C&D). Increased Ly6G expression in B6.CCR7^-/-^ splenic monocytes indicated a portion of neutrophilic cells (Figure 2E). Population 2 (Ly6C^int^) had increased Ly6G expression and could be identified as neutrophilic in the lesion and spleen (Figure 2E). The proinflammatory capability of population 2 could be decreased in B6.CCR7^-/-^ mice compared to B6.WT mice as shown by their lower CCR2 and iNOS expression (Figure 2C&D). Finally, the Ly6C^hi^ population (population 3) showed high CCR2 expression and was consequently identified as inflammatory monocytes (Figure 2C). Furthermore, the proportion of Ly6C^hi^ cells was increased in the footpad of B6.CCR7^-/-^ mice (Figure 2B).

**Figure 3:**
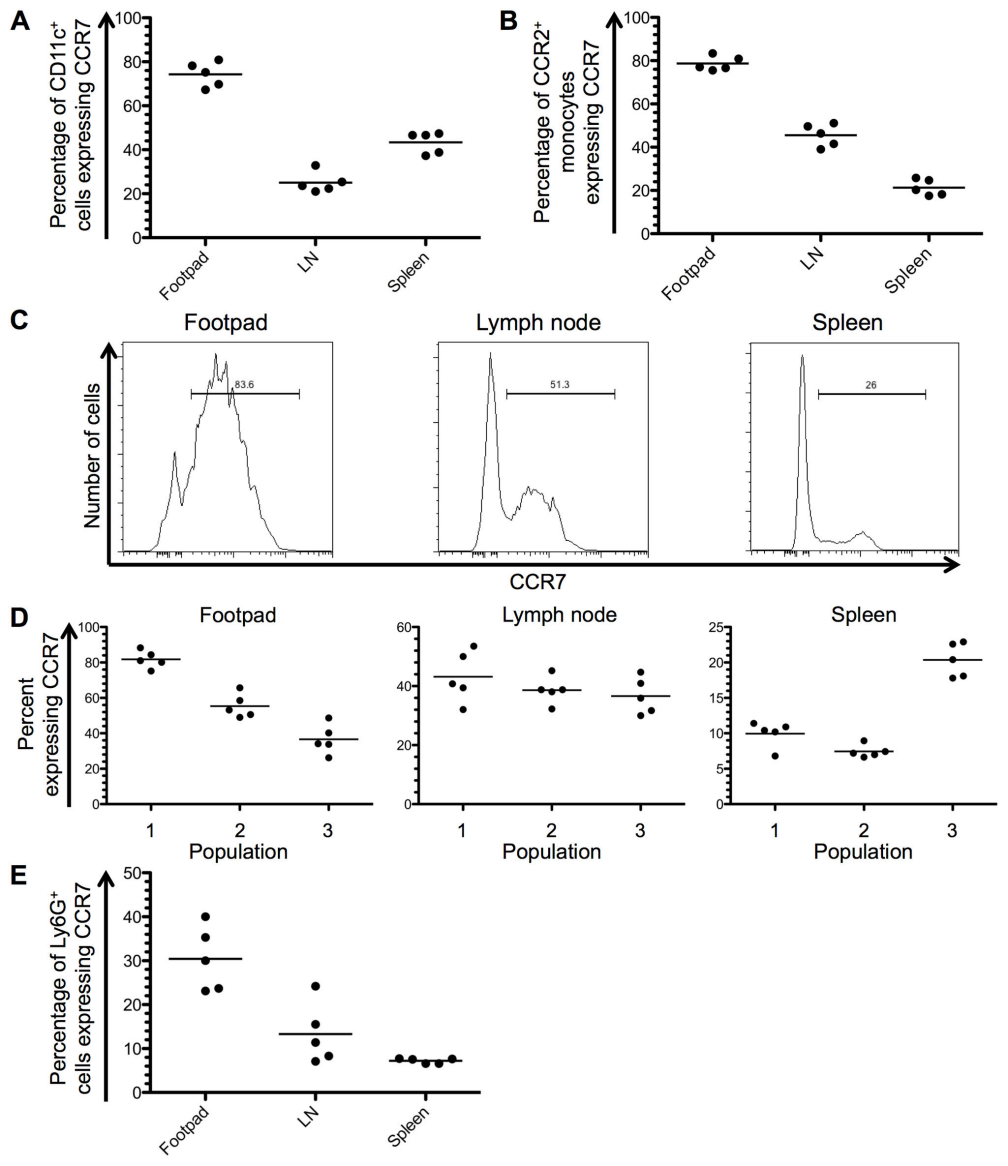
CCR7 expression in B6.WT mice at day 14 after infection with *L. major*. (A) The percentage of CD11c^+^ cells expressing CCR7 was determined by flow cytometry in the footpad, draining lymph node (LN) and spleen. (B) The expression of CCR7 on CD11b^+^CCR2^+^ monocytes was determined in each tissue of B6.WT mice. (C) A representative histogram of CCR7 expression on CD11b^+^CCR2^+^ monocytes in each tissue is shown. (D) Monocytic populations were defined according to their expression of Ly6C as shown in Figure 2A, and the percentage of cells in each population expressing CCR7 is shown. (E) The percentage of Ly6G^+^ neutrophils in each tissue expressing CCR7 was analysed by flow cytometry. Experimental group size: n=5 mice.

At day 14 after infection, the percentage of CD11c^+^ cells expressing CCR7 in B6.WT mice was 74.3% (±2.5 [SEM]) in the footpad, 25.1% (±2.1) in the lymph node and 43.3% (±2.2) in the spleen (Figure 3A). In the footpad, 78.7% (±1.5) of CCR2^+^ monocytes also expressed CCR7, while 45.5% (±2.3) and 21.3% (±1.7) of CCR2^+^ cells were CCR7^+^ in the lymph node and spleen, respectively (Figure 3B&C). According to the populations described in Figure 2A, a larger proportion of population 1 (Ly6C^lo^) in the footpad expressed CCR7. There was no difference in expression between the three populations in the lymph node (Figure 3D), while in the spleen, population 3 (Ly6C^hi^) had a greater percentage of cells expressing CCR7. The expression of CCR7 was also analysed on Ly6G^+^ cells [21], which were confirmed by flow cytometry to be neutrophilic due to their high side scatter profile. In the footpad, 30.4% (±3.3) of Ly6G^+^ cells were CCR7^+^, while in the lymph node 13.3% (±3.1) and in the spleen 7.2% (±0.25) were also CCR7^+^ (Figure 3E).

**Figure 4:**
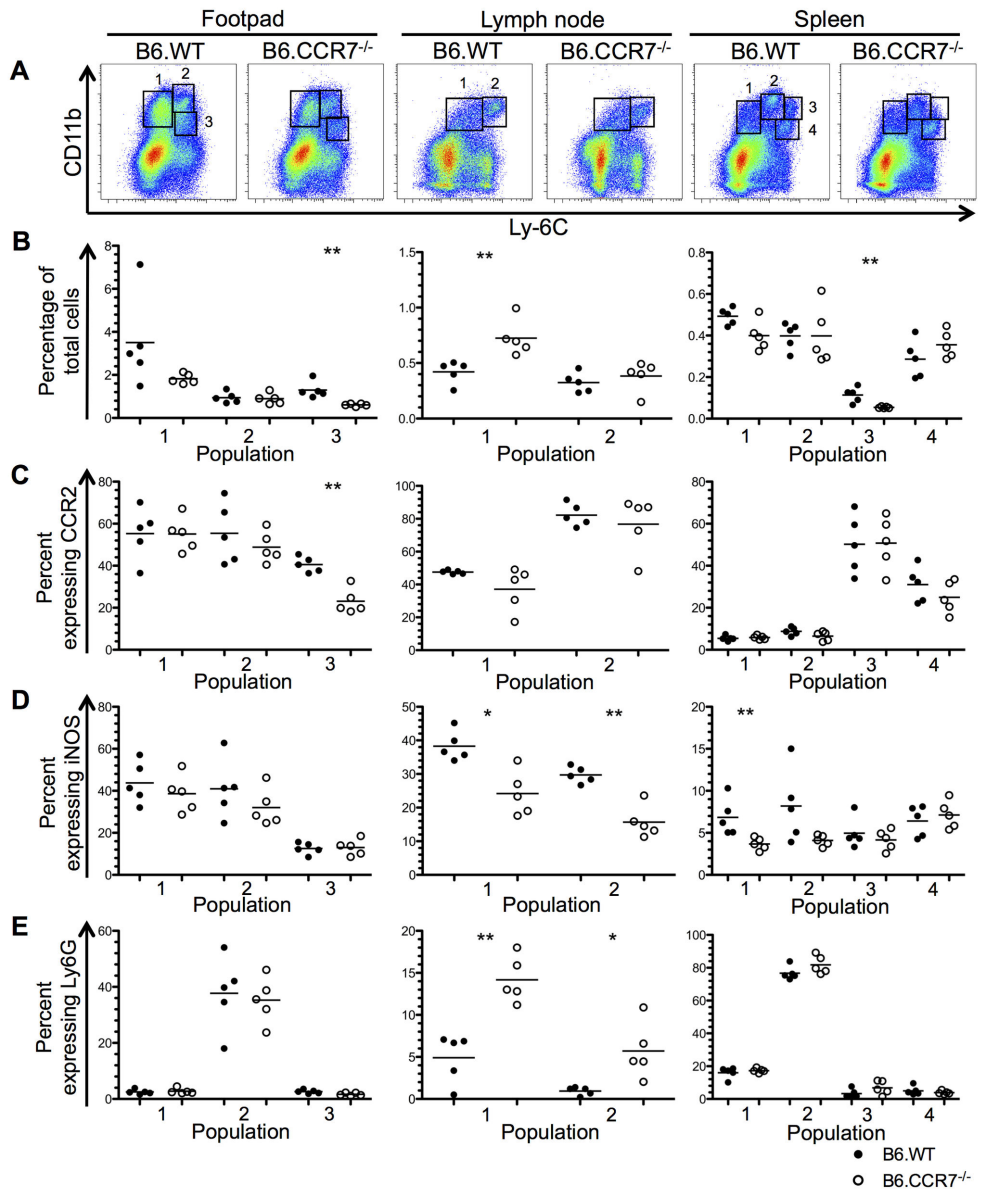
Monocytic populations in B6.WT and B6.CCR7^-/-^ mice at day 28 after infection with *L. major*. (A) Flow cytometry was used to determine monocytic populations in the footpad, lymph node and spleen according to their CD11b and Ly6C expression. (B) The percentage of the total cell number recovered from each tissue is shown for B6.WT (closed circles) and B6.CCR7^-/-^ (open circles) mice for each monocytic population. The percentage of cells in each population expressing CCR2 (C), iNOS (D) and Ly6G (E) was determined. One representative analysis of three experiments; Experimental group size: n=5 mice/genotype; *p<0.05; **p<0.01.

By day 28 after infection, the monocytic populations had differentiated further and formed three populations in the footpad, two in the lymph node and four in the spleen (Figure 4A). In the footpad, population 1 appeared similar in both genotypes in terms of CCR2, iNOS and Ly6G expression (Figure 4C-E). Population 2 was neutrophilic as demonstrated by its increased Ly6G expression (Figure 4E) and the percentage of the previously CCR2^+^ inflammatory monocytes (population 3) was significantly reduced in B6.CCR7^-/-^ mice, and showed only a fraction of previous CCR2 expression (Figure 4B&C). In the lymph node of B6.CCR7^-/-^ mice, the percentage of non-inflammatory monocytes in population 1 (Ly6C^lo^) had increased, while the previous inflammatory population 2 (Ly6C^hi^) displayed a more neutrophilic phenotype as demonstrated by decreased iNOS and increased Ly6G expression (Figure 4D&E). In the spleen of B6.CCR7^-/-^ mice, population 1 showed decreased iNOS expression compared to the B6.WT subset (Figure 4D). Population 2 expressed Ly6G, and resembled neutrophils (Figure 4E) while populations 3 and 4 had increased CCR2 expression, which was indicative of inflammatory monocytes (Figure 4C). 

**Figure 5:**
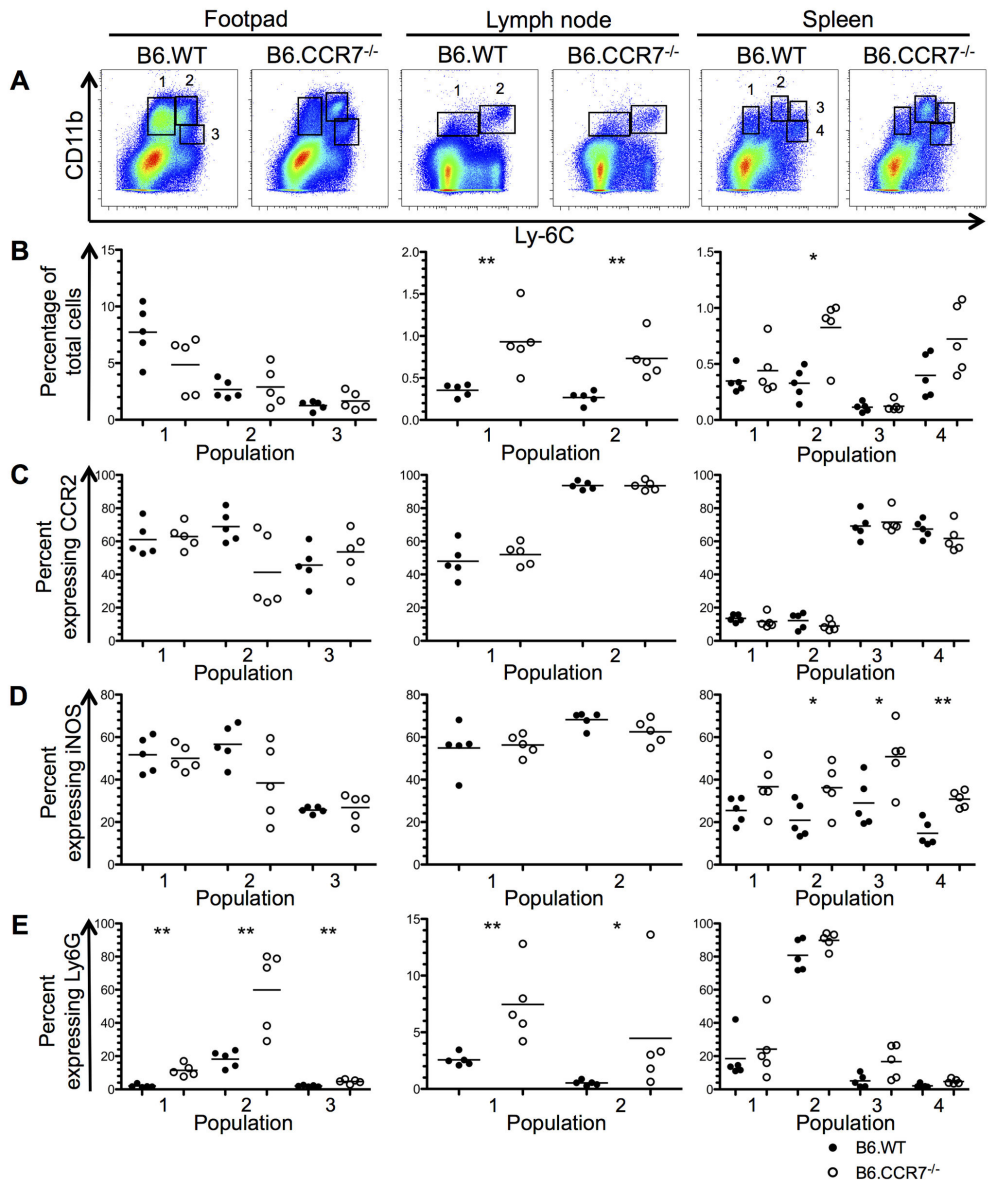
Characterisation of monocytic populations in B6.WT and B6.CCR7^-/-^ mice at day 42 post infection. (A) Populations of monocytes in each tissue were defined by flow cytometry according to their CD11b and Ly6C expression. (B) The percentage of these populations in each tissue is shown in B6.WT (closed circles) and B6.CCR7^-/-^ (open circles) mice. The expression of CCR2 (C), iNOS (D) and Ly6G (E) was determined by flow cytometry and displayed as a percentage of each population. One representative analysis of three experiments; Experimental group size: n=5 mice/genotype; *p<0.05; **p<0.01.

At day 42 after infection, the monocytic populations appeared similar to the subpopulations detected on day 28 (Figure 5A). The lymph nodes of B6.CCR7^-/-^ mice had increased percentages of both monocytic populations (Figure 5B). There was also increased iNOS expression by monocytes in the spleen of B6.CCR7^-/-^ mice (Figure 5D). Overall, at this timepoint, the inflammatory infiltrate in all tissues of B6.CCR7^-/-^ mice was composed of Ly6G^+^ neutrophils (Figure 5E), rather than CCR2^+^ inflammatory monocytes (Figure 5C).

### Monocyte and neutrophil migration is dysregulated within the spleen and lymph node of B6.CCR7^-/-^ mice

**Figure 6:**
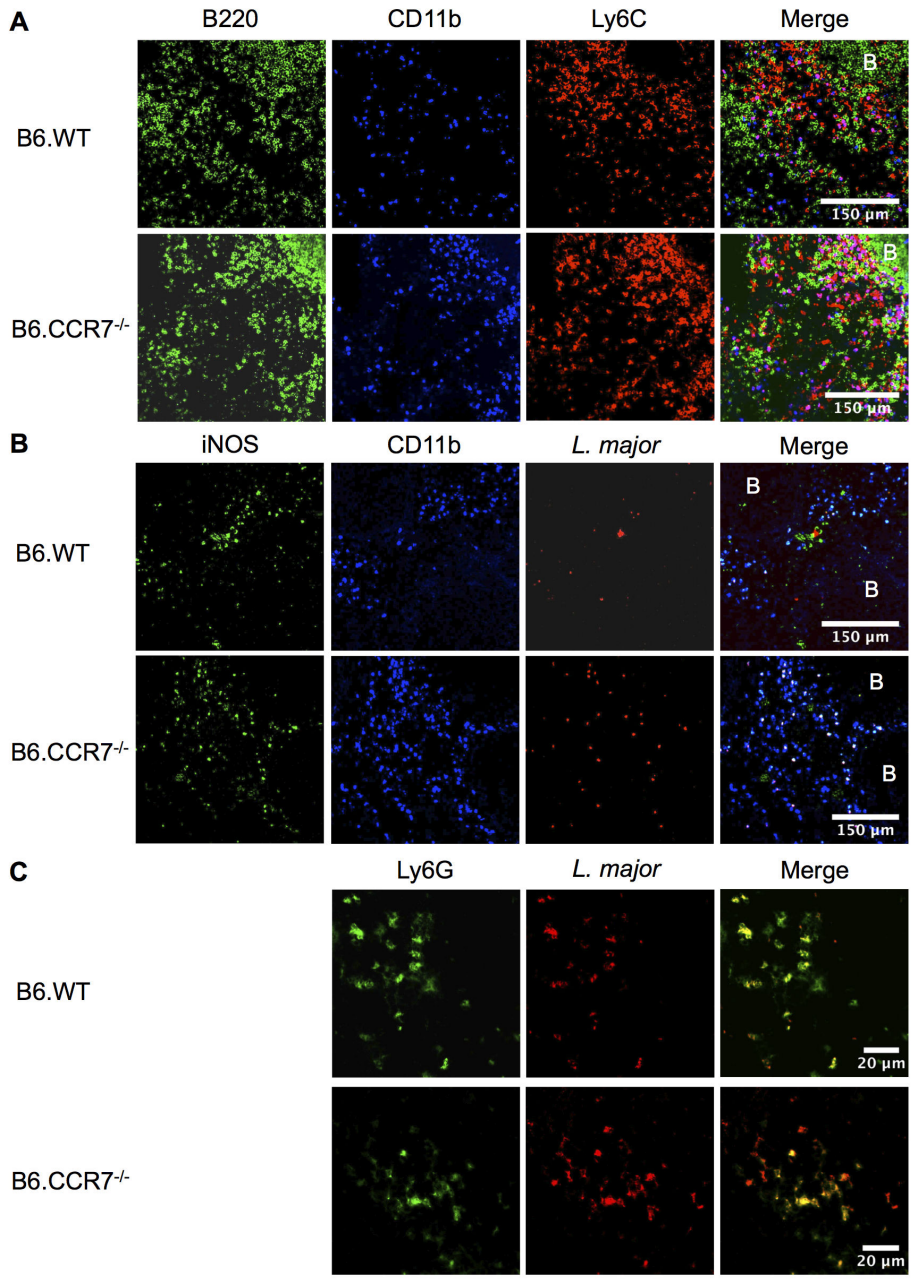
Fluorescent immunohistology of B6.WT and B6.CCR7^-/-^ spleens at day 14 post infection and lymph nodes 24 hours after infection. (A) Frozen spleens from day 14 post infection were sectioned and stained with antibodies against B220 (green), CD11b (blue) and Ly6C (red) to show localisation of monocytes in the spleen. (B) Expression of iNOS (green), CD11b (blue) and *L. major* (red) was shown in the spleen. (C) The draining popliteal lymph node was taken at 24 hours after infection with *L. major* expressing eGFP (red) and analysed for Ly6G (green) expression. B=B cell follicle; Objective magnification: A&B 20x; C 63x.

Based on the flow cytometry data, we analysed the structure of the spleens at day 14 after infection. Tissue was sectioned and stained for the *in situ* location of monocytes, neutrophils and *L. major*. In B6.CCR7^-/-^ spleens, most CD11b^+^ cells were also Ly6C^+^ (Figure 6A) indicating the dominant presence of inflammatory monocytes (Ly6C^hi^) or neutrophils (Ly6C^lo^). In contrast, the majority of CD11b^+^ cells in B6.WT mice did not express Ly6C^+^ (Figure 6A). Histologically, in B6.WT mice, these CD11b^+^Ly6C^+^ cells were excluded from the B220^+^ B cell follicle, while in B6.CCR7^-/-^ mice, CD11b^+^Ly6C^+^ cells appeared to be located in all compartments of the spleen, including the B cell follicle (Figure 6A). While *L. major* parasites are visible in clusters in B6.WT mice [19] they were distributed throughout the spleen of B6.CCR7^-/-^ mice (Figure 6B). In B6.WT mice, both myeloid (CD11b^+^) and non-myeloid (CD11b^-^) cells expressed iNOS, whereas in B6.CCR7^-/-^ mice, the parasites and iNOS expression was co-localised within CD11b^+^ cells. The parasite burden of neutrophils was specifically analysed in the draining lymph node 24 hours after infection (Figure 6C). Neutrophils harboured *L. major* parasites in both B6.WT and B6.CCR7^-/-^ mice. While parasites could only be found in neutrophils in B6.WT mice after 24 hours post infection, other cells had taken up parasites in B6.CCR7^-/-^ lymph nodes (Figure 6C). Taken together, the monocytes are not confined to their usual compartments in B6.CCR7^-/-^ mice and may interact with other cells that would otherwise not be in proximity with each other.

### Increased IL-12, but not IFN-γ, and increased IL-4 after infection in B6.CCR7^-/-^ lymph nodes

**Figure 7:**
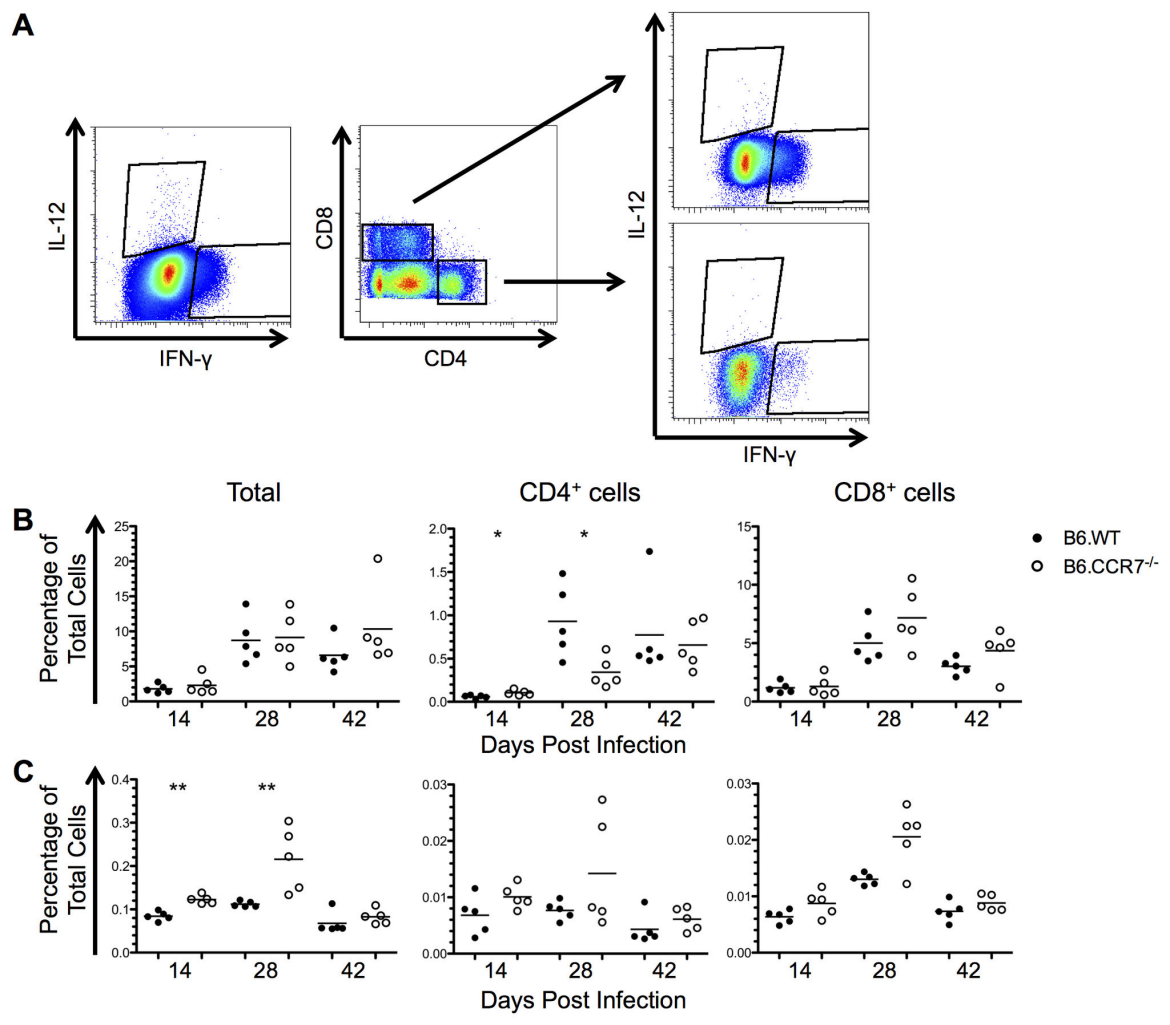
Cellular expression of Th1 cytokines in the lymph node of mice infected with *L. major*. (A) Intracellular expression of IL-12 and IFN-γ was determined by flow cytometry. (B) The expression of IFN-γ in B6.WT (closed circles) and B6.CCR7^-/-^ (open circles) mice is shown as a percentage of all lymph node cells as well as a the percentage of CD4^+^ or CD8^+^ cells expressing IFN-γ. (C) The expression of IL-12 was shown in a similar manner to IFN-γ. One representative analysis of three experiments; Experimental group size: n=5 mice/genotype for each timepoint; *p<0.05; **p<0.01.

Intracellular flow cytometry was employed to analyse cytokine expression on a cellular basis. Cells were gated for the cytokine and CD4 or CD8 expression (Figure 7A). The proportion of CD4^+^IFN-γ^+^ T cells was increased significantly in B6.CCR7^-/-^ mice as compared to B6.WT mice at day 14 after infection (Figure 7B). By day 28 post infection both genotypes displayed an increased number of CD4^+^IFN-γ^+^ T cells, which was more pronounced in B6.WT mice (Figure 7B). However, IFN-γ was predominately produced by CD8^+^ T cells in equal proportions in both B6.WT and B6.CCR7^-/-^ mice. The level of IL-12 peaked at day 28 in B6.CCR7^-/-^ mice, while they remained relatively stable in B6.WT mice (Figure 7C). Furthermore, B6.CCR7^-/-^ mice had a greater percentage of cells producing IL-12 at days 14 and 28 post infection than B6.WT mice (Figure 7C) and most of this production was by cells other than T cells.

**Figure 8:**
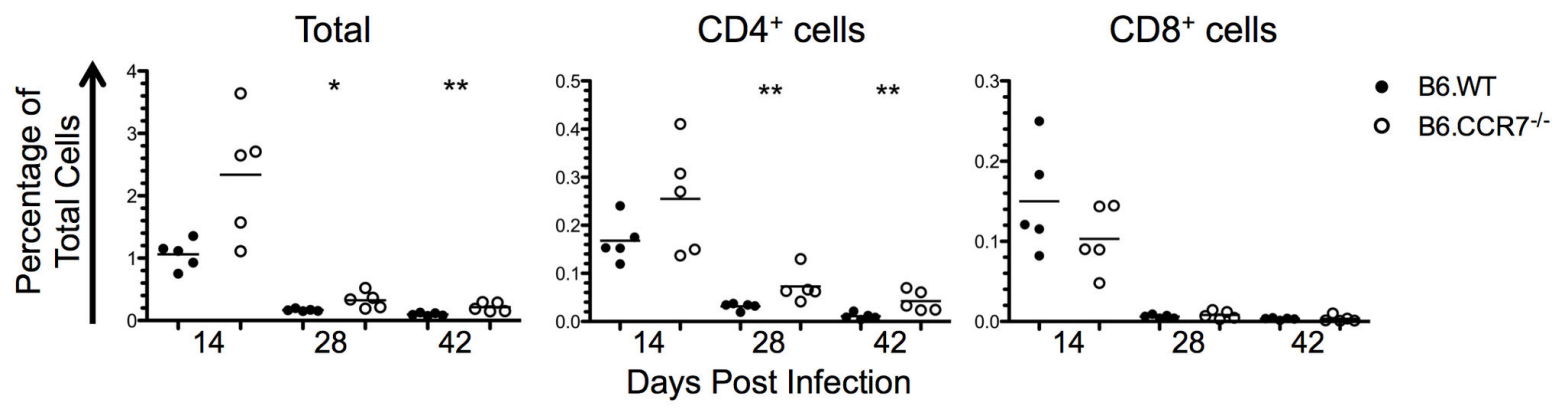
Expression of the Th2 cytokine, IL-4, in the lymph node of infected mice. The percentage of all lymph node leukocytes as well as the percentage of CD4^+^ or CD8^+^ cells expressing IL-4 was determined by intracellular flow cytometry in B6.WT (closed circles) and B6.CCR7^-/-^ (open circles) mice. One representative analysis of three experiments; Experimental group size: n=5 mice/genotype for each timepoint; *p<0.05; **p<0.01.

The Th2 cytokine, IL-4, which is present in susceptible mice [22], was also analysed. Initially, at day 14 post infection, there is a large percentage of cells expressing IL-4, which then decreased at days 28 and 42 post infection (Figure 8). Furthermore, at days 28 and 42 post infection, B6.CCR7^-/-^ mice have a higher percentage of cells expressing IL-4, which was also seen in the CD4^+^ compartment but not by CD8^+^ T cells. Other cells, such as basophils and mast cells, contribute to the excess IL-4 production, not provided by T cells [23,24].

### Increased immunosuppression in lymph nodes lacking CCR7

**Figure 9:**
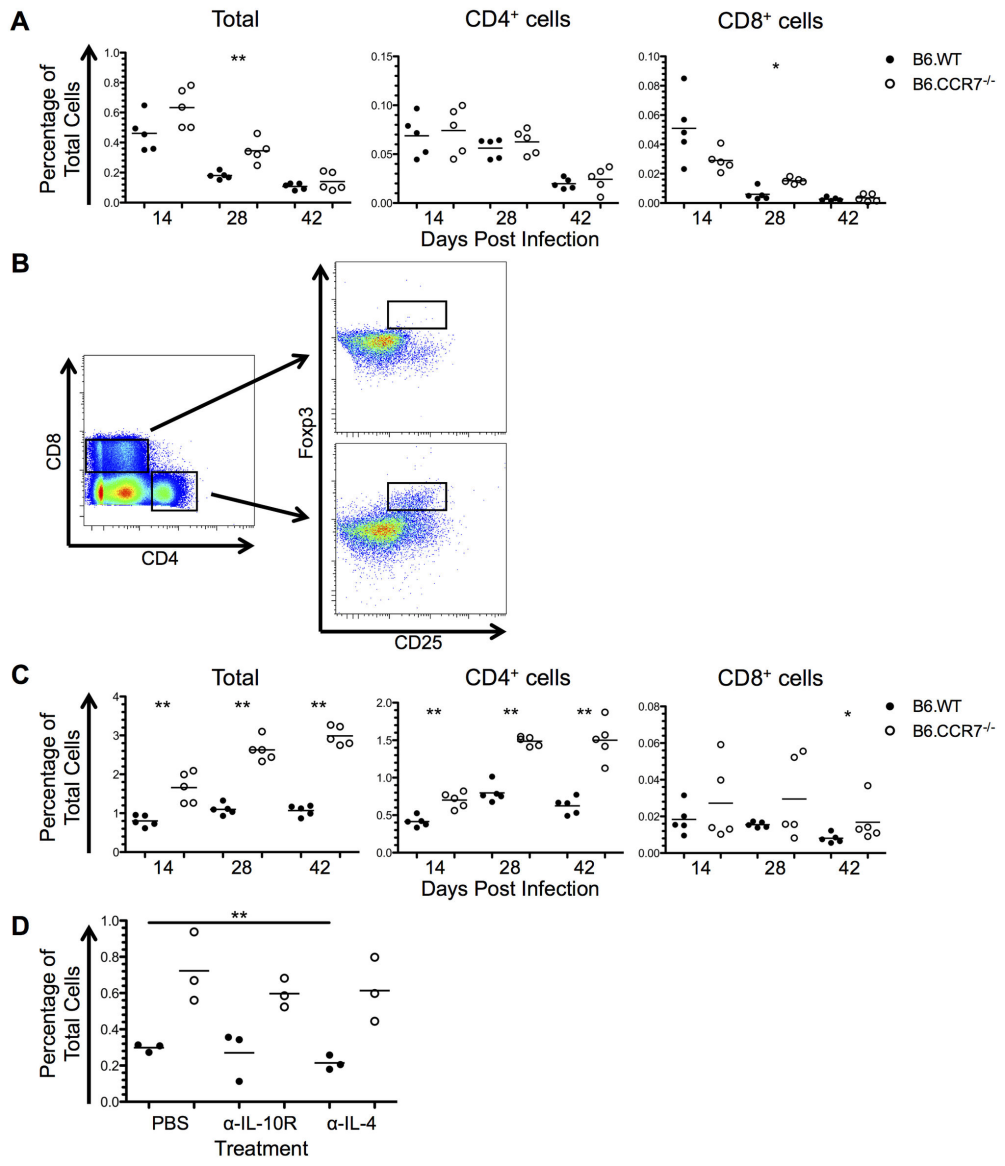
Immunosuppression in the lymph nodes of B6.WT and B6.CCR7^-/-^ during infection with *L. major*. (A) Expression of IL-10 in the total lymph node, as well as in the CD4^+^ and CD8^+^ compartments was determined by flow cytometry in B6.WT (closed circles) and B6.CCR7^-/-^ (open circles) mice. (B) CD4^+^ or CD8^+^ Tregs were determined by their dual expression of CD25 and Foxp3. (C) The total percentage of Foxp3^+^ cells as well as the percentage of CD4^+^ and CD8^+^ Tregs in the total population was determined by intracellular flow cytometry. (D) Mice were injected intraperitoneally with PBS, anti-IL-10R or anti-IL-4, and the draining lymph node analysed for the percentage of CD4^+^ Tregs at day 14 post infection. A&C, One representative analysis of three experiments; Experimental group size: n=5 mice/genotype for each timepoint; D, One experimental analysis; Experimental group size: n=3 mice/genotype for each treatment. *p<0.05; **p<0.01.

The expression of IL-10 and the transcription factor Foxp3 was analysed to determine the immunosuppressive capability of B6.CCR7^-/-^ mice. The percentage of IL-10 producing cells decreased throughout infection in both genotypes (Figure 9A). At day 28 post infection there was an increase in IL-10-producing cells in B6.CCR7^-/-^ mice, which was also seen in CD8^+^ cells. Some of the IL-10 production is by cells other than T cells, and could be the result of monocytic or dendritic cell expression [25].

Regulatory T cells (Tregs) were recognised by the expression of both CD25 and Foxp3 (Figure 9B). Throughout the infection there was an increased proportion of CD4^+^ cells expressing Foxp3 in B6.CCR7^-/-^ mice (Figure 9C). Additionally, there was some Foxp3 expression by CD8^+^ cells, which increased significantly at day 42 post infection in B6.CCR7^-/-^ mice (Figure 9C). These CD8^+^Foxp3^+^ T cells have not yet been described functionally in parasitic diseases, but could represent cells with suppressive ability [26]. While TGF-β and IL-2 promote the differentiation of Tregs, TGF-β and IL-6 induce Th17 differentiation and inhibit the development of Tregs [27]. Due to the reciprocal developmental pathway, Th17 cells (CD4^+^IL-17^+^) were also analysed, and an increased percentage was seen only at day 28 after infection in B6.CCR7^-/-^ mice (data not shown).

Since there were increased Tregs present in B6.CCR7^-/-^ mice, the mechanism behind this accumulation was analysed. Due to the observation of an increase in the Th2 and immunosuppressive cytokines IL-4 and IL-10, we explored the possibility that these cytokines played a role in promoting Treg differentiation in B6.CCR7^-/-^ mice. We therefore injected mice for two weeks with a blocking anti-IL-10R antibody or a neutralising anti-IL-4 antibody in an attempt to decrease the percentage of Tregs. While the percentage of Tregs in the lymph node decreased in B6.WT mice injected with an anti-IL-4 antibody compared to control B6.WT mice injected with PBS, the percentage of Tregs remained unchanged in B6.CCR7^-/-^ mice, irrespective of antibody treatment (Figure 9D). This data show that the increase in Tregs in the lymph nodes of B6.CCR7^-/-^ mice is independent of IL-10 or IL-4 signalling.

Overall, there is an early increase in the macrophage-activating Th1 cytokines IFN-γ and IL-12, followed by an increase in Th2 and immunosuppressive cytokines from the peak of disease into the chronic stage of infection in B6.CCR7^-/-^ mice. The cytokine production by cellular sources correlated with the systemic production of proinflammatory and immunosuppressive cytokines in the serum (Figure S2). Furthermore, there is an increased percentage of Tregs throughout infection. The skewing of the immune response to a detrimental Th2 and immunosuppressive response at the late stages may contribute to the inability to clear the parasites from B6.CCR7^-/-^ mice and cause a chronic infection.

## Discussion

The chemokine receptor CCR7 has been studied comprehensively in homeostasis and inflammation [7]. This extensive body of work demonstrates, for example, a clearly defined role of CCR7 in the homing of naïve T cells to lymph nodes [14]. However, an analysis of the function of this receptor in complex infection models shows that there is tolerance, allowing cell migration in the absence of CCR7 under inflammatory conditions [8,10]. We sought to determine the effects of the absence of CCR7 on the resolution of leishmaniasis, as well as the composition and function of the inflammatory infiltrate. After subcutaneous infection with *L. major*, B6.CCR7^-/-^ mice were unable to resolve the lesion and developed a chronic infection. Furthermore, we found a significantly increased number of neutrophils present in the infected footpad, draining lymph node and spleen, as well as delayed migration of CCR2^+^ inflammatory monocytes. A further analysis of CCR2^+^ monocytes showed significant CCR7 expression. Finally, we observed that the balance of helper T cell cytokines in the lymph node was skewed towards a Th2 phenotype, and we detected an increased percentage of Tregs throughout infection.

In an infection with viral or bacterial agents, which require a host immune response dominated by cytotoxic CD8^+^ T cells for clearance of the pathogen, CCR7^-/-^ mice display a delayed, but ultimately protective response [8,10]. However, infection of B6.CCR7^-/-^ mice with the parasite *Toxoplasma gondii*, which requires a CD4^+^ Th1 response, results in death during the acute phase of the disease due to uncontrolled parasite replication and dissemination [11]. In our experiments, infection of B6.CCR7^-/-^ mice with *L. major*, which is also eliminated by a protective Th1 response [28,29], does not result in death, but the mice are still unable to clear the parasite, and suffer from a chronic infection (Figure 1A). Together, these studies during infection demonstrate that the functions of CCR7 are largely redundant for cytotoxic T cell responses but indicate that they are critical for Th1 responses against intracellular parasites. This could be due to a requirement for CCR7-dependent migration of cells that present antigen via MHC class II to CD4^+^ T cells, while MHC class I restricted presentation to CD8^+^ T cells relies only in part on the migration of cells to lymphoid organs [14,30].

Inflammatory monocytes expressing the chemokine receptor CCR2 are critical for the clearance of intracellular parasites by antigen presentation and by utilising a range of effector mechanisms, such as iNOS [19,31–35]. While it is well established that the presence of CCR2^+^ monocytes is essential for the elimination of both *T. gondii* and *L. major* from the site of infection [19,31], the influence of other chemokine receptors, such as CCR7, on the migration of these cells has not been explored in more detail, despite indirect evidence of delayed migration in the absence of CCR7. Similar to our *L. major* model, CCR7^-/-^ mice infected with *T. gondii* show a reduced migration of CCR2^+^ inflammatory monocytes to the site of infection [11]. This supports evidence that CCR7 is required for the migration of monocytes into inflamed tissue, while the role of CCR2 seems to be limited to migration from the bone marrow to the circulation [36,37]. For the first time in an infection model, we have shown that the majority of CCR2^+^ monocytes in the footpads of B6.WT mice also express CCR7 (Figure 3B). However, it is unknown if these monocytes have recently been recruited to the site of infection, or if they have upregulated CCR7 in preparation for migration to the draining lymph node.

The CCR7-dependent migration of cells depends on the tissue-specific gradients of its ligands [3]. In mice, CCR7 has two ligands, CCL19 and CCL21, with CCL21 displaying two isoforms differing in one amino acid, CCL21-Ser and CCL21-Leu [1,38]. The expression of the chemokines CCL19 and CCL21 is usually associated with lymphoid organs, nevertheless, CCL21-Leu is also constitutively expressed in non-lymphoid organs, and CCL21-Ser can be upregulated within lung tissue upon inflammation [38,39]. Furthermore, in a visceral leishmaniasis model, CCL21 expression correlates with parasite burden in the dermis of infected dogs [40]. Combined with the ability of CCL21 to be expressed in non-lymphoid tissues and the delayed migration of inflammatory monocytes to the footpad in the absence of CCR7, it could be speculated that CCR7, rather than CCR2, is required for the inflammatory recruitment of monocytes to the site of infection [11].

The immune response to *Leishmania*, like any other infection, is shaped by the presence of Tregs [41]. Specifically, in the model of cutaneous leishmaniasis, Tregs prevent complete eradication of the parasite from the host through IL-10 mediated mechanisms [42]. Chemokine receptors, including CCR7, are known to be important for the migration and function of Tregs [43,44]. In wild type mice, CCR7 is present on a subset of Tregs that do not express αE integrin (CD103), a molecule involved in the detainment of Tregs at the site of infection [45–47]. Consequently, these Tregs home to lymph nodes, whereas in the absence of CCR7, there is retention of Tregs at inflamed sites [45,48]. More specifically, while CCR7^-/-^ Tregs are suppressive *in vitro*, these cells are less efficient *in vivo* after ovalbumin sensitisation, due to impaired migration to lymphoid tissues and incorrect localisation within the target lymphoid organs [45,46,49]. This is of particular relevance for *L. major* infection, since CD4^+^CD25^+^ Tregs prevent a sterile cure, and therefore, there is potential for reactivation of leishmaniasis [41,42,50]. In our model, *L. major* infection causes an increase in the percentage of Tregs in the draining lymph node (Figure 9C), as well as the infected footpad (data not shown) in the absence of CCR7, potentially contributing to the increased parasite burden and chronically enlarged lesion [42].

The model of cutaneous leishmaniasis has been beneficial in the elucidation of the Th1/Th2 paradigm [51]. A Th1 response is characterised by IFN-γ and IL-12 expression, while a Th2 response is characterised by IL-4 production, and the T cell responses are associated with resistance or susceptibility, respectively [22]. The genetic background of C57BL/6 mice is able to mount a protective response, but in infected B6.CCR7^-/-^ mice, the T cell response was skewed towards a susceptible Th2 phenotype. The skewing of the immune system towards a Th2 phenotype in B6.CCR7^-/-^ mice has also been shown using an ovalbumin sensitisation model [52]. However, despite an increase in IL-4 expression, these CCR7-deficient mice do not develop a progressive disease, but instead develop a chronic infection. This supports the notion that the mechanisms responsible for resistance only rely in part on cytokine production. Recent studies have shown that the wound healing response can also play a role in resistance or susceptibility to the parasite and this response is characterised by collagen deposition [53,54]. In the skin, several different cell types contribute to wound healing, such as fibrocytes and mesenchymal stem cells. In an interesting coincidence, CCR7 and its ligands have also been shown to be centrally involved in wound healing [55–58]. In pulmonary fibrosis, a model of exacerbated wound healing, the absence of CCR7 alleviates the disease, and in a skin injury model, fibrocytes are able to migrate to wound sites and enhance healing in a CCR7-dependent manner [59,60]. Furthermore, mesenchymal stem cells are able to migrate to skin wounds in response to CCR7 ligands and support wound healing [57]. Since we showed that B6.CCR7^-/-^ mice have an enlarged lesion for an extended period of time, it could be speculated that the wound healing process is inefficient in these mice. Our data indicate that the CCR7-deficient infection model may be beneficial to address other mechanisms of susceptibility and resistance to *L. major* infection, such as the wound healing response.

In summary, the chemokine receptor CCR7 plays a role in the migration of inflammatory monocytes and Tregs. Disruption of the normal function and migration of these cells in the absence of CCR7 during cutaneous leishmaniasis causes a chronic infection. Our data further demonstrates that the role of CCR7 is extremely complex in infection models, and more studies are required to further elucidate the mechanisms of action between CCR7 and its ligands.

## Supporting Information

Figure S1 **Representative flow cytometric analysis of monocytes.** Representative staining of splenic samples from day 28 post infection are shown for CCR2 expression (A), iNOS expression (B) and Ly6G expression (C) within each monocytic population described in Figure 4.
(TIFF)

Figure S2 **Cytokine levels in the serum of B6.WT and B6.CCR7^-/-^ mice throughout infection with *L. major*.** Cytometric bead arrays were used to determine the levels of (A) IFN-γ, (B) IL-12p70, (C) IL-1β, (D) TNF, (E) IL-4 and (F) IL-10 in the serum of infected mice at days 14, 28 and 42 post infection. Experimental group size: n=5 mice/genotype for each timepoint; ND=not detectable.
(TIFF)
