# Supplementary figures and images for: The Absence of CCR7 Results in Dysregulated Monocyte Migration and Immunosuppression Facilitating Chronic Cutaneous Leishmaniasis
Source: PLoS One. 2013 Oct 30;8(10):e79098. doi: 10.1371/journal.pone.0079098 (PMC3813618; doi:10.1371/journal.pone.0079098)

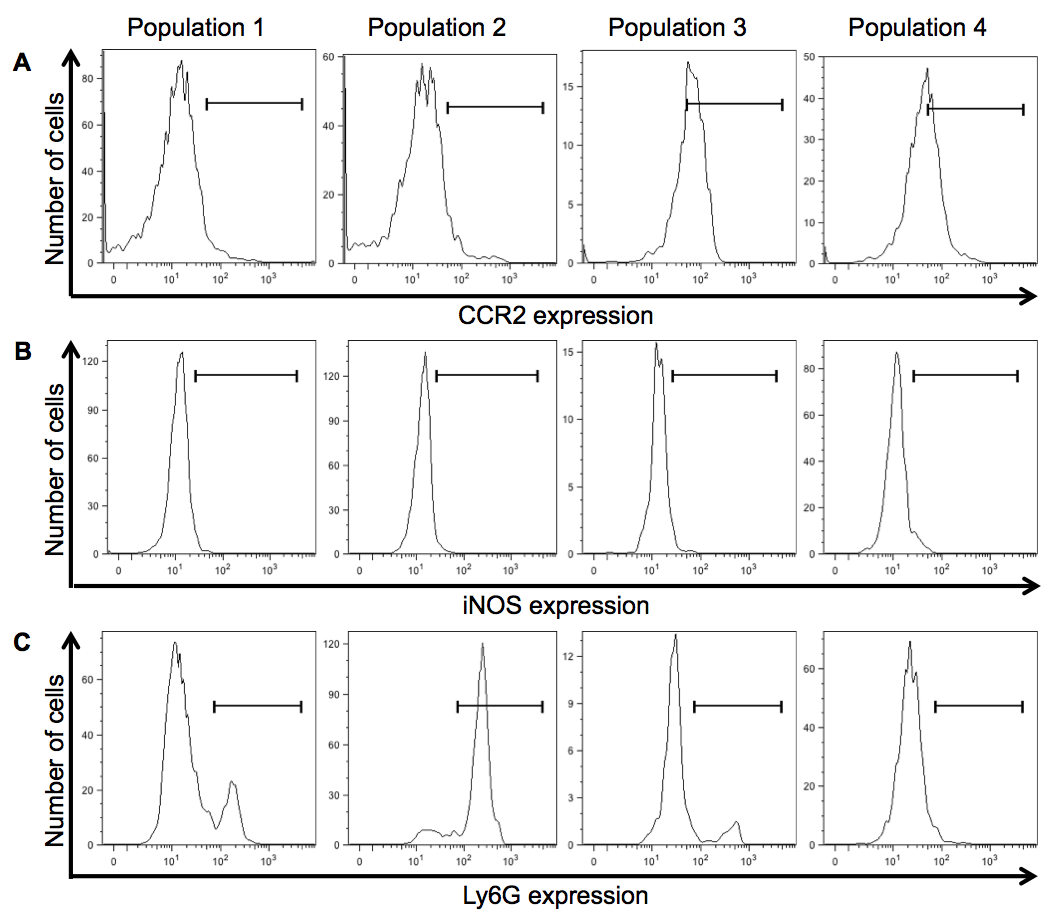

Supplement: Figure S1 — Representative flow cytometric analysis of monocytes. Representative staining of splenic samples from day 28 post infection are shown for CCR2 expression (A), iNOS expression (B) and Ly6G expression (C) within each monocytic population described in Figure 4. (TIFF) [file pone.0079098.s001.tiff]

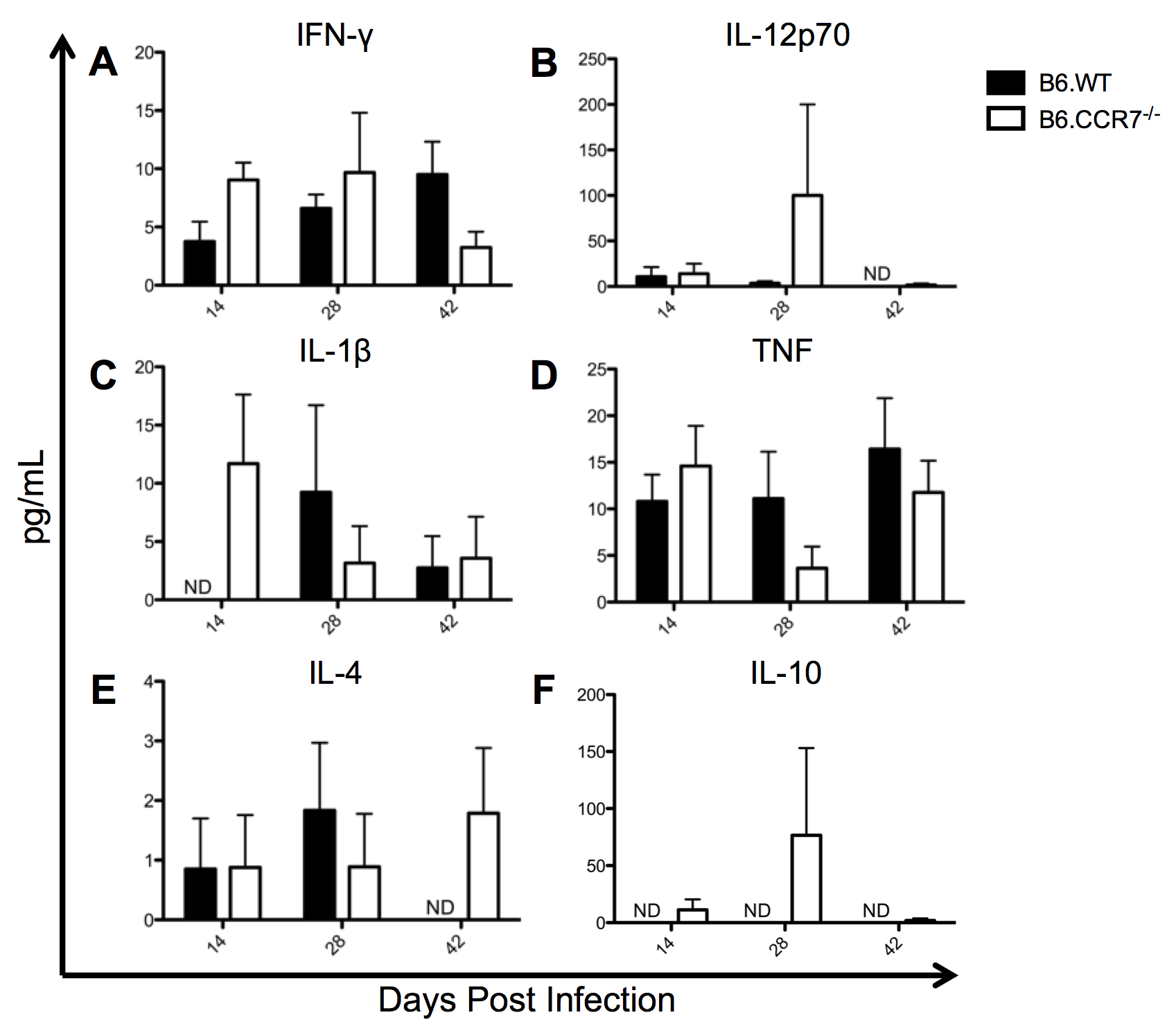

Supplement: Figure S2 — Cytokine levels in the serum of B6.WT and B6.CCR7-/- mice throughout infection with L. major. Cytometric bead arrays were used to determine the levels of (A) IFN-γ, (B) IL-12p70, (C) IL-1β, (D) TNF, (E) IL-4 and (F) IL-10 in the serum of infected mice at days 14, 28 and 42 post infection. Experimental group size: n=5 mice/genotype for each timepoint; ND=not detectable. (TIFF) [file pone.0079098.s002.tiff]
